# Nitrate-reducing microorganisms prevent souring of an oil field produced water storage pond

**DOI:** 10.1093/femsec/fiaf041

**Published:** 2025-04-28

**Authors:** Gabrielle Scheffer, Jayne Rattray, Paul Evans, Wei Shi, Srijak Bhatnagar, Casey R J Hubert

**Affiliations:** Geomicrobiology Group, Department of Biological Sciences, University of Calgary, Calgary, Alberta T2N 1N4, Canada; Geomicrobiology Group, Department of Biological Sciences, University of Calgary, Calgary, Alberta T2N 1N4, Canada; Chevron Technical Center, Houston, Texas 77072, United States; Chevron Technical Center, Houston, Texas 77072, United States; Faculty of Science and Technology, Athabasca University, Athabasca, Alberta T9S 3A3, Canada; Geomicrobiology Group, Department of Biological Sciences, University of Calgary, Calgary, Alberta T2N 1N4, Canada

**Keywords:** autotrophic microorganisms, hydraulic fracturing, nitrate-reducing microorganisms, produced water reuse, storage ponds, sulfide-oxidizing microorganisms

## Abstract

Nitrate addition for mitigating sulfide production in oil field systems has been studied in laboratory settings and in some subsurface oil reservoirs. To promote water recycling and reuse associated with oil reservoirs produced by hydraulic fracturing, high-salinity produced waters are temporarily stored in surface ponds prior to subsequent reinjection into the subsurface. In this study, nitrate was added directly to a storage pond to prevent sulfide accumulation. DNA sequencing of pond water over a 4-week period revealed a decrease in the proportion of sulfate-reducing microorganisms following nitrate application. Sulfate levels remained stable during this period, whereas nitrate and nitrite fluctuated in the days following the nitrate addition. Metagenome-assembled genomes (MAGs) reconstructed from the pond water microbiome highlighted different organisms with genes for organoheterotrophic and lithoheterotrophic nitrate reduction, whereas genes associated with sulfide production via sulfate or thiosulfate reduction were barely detected. Within those MAGs, genes for acetate metabolism were observed, consistent with acetate decreasing substantially in the pond water in the presence of nitrate. After nitrate was consumed an increase in relative abundance of putative autotrophic microorganisms was observed (e.g. *Arhodomonas, Guyparkeria*, and *Psychroflexus*), corresponding to a drop in total inorganic carbon measurements in the storage pond. This trial offers an overview on microbial processes taking place in storage pond environments in response to nitrate addition.

## Teaser

Nitrate was added to a storage pond to control potential souring of a storage pond to reuse during the hydraulic fracturing process.

## Introduction

The presence and influence of microorganisms in oil fields can be detrimental in many instances related to biofouling, undesired plugging, microbially influenced corrosion, and souring (Enning and Garrelfs 2014, Basafa and Hawboldt 2019, Nicoletti et al. 2022). Certain microbial populations can also be beneficial in these settings and offer cost-effective solutions to different problems. Of particular interest is controlling sulfide production (souring) by adding nitrate to stimulate nitrate-reducing microorganisms (NRM) that combat the activity of sulfate-reducing microorganisms (SRM) and other sulfidogens (Hubert and Voordouw 2007, Kumaraswamy et al. 2011, An et al. 2017, Suri et al. 2017, Carlson and Hubert 2019). Oxidation of organic or inorganic electron donors coupled to nitrate reduction by organotrophic or lithotrophic NRM is more thermodynamically favorable than coupling the same oxidative processes to sulfate reduction; hence, nitrate injection can block the activity of SRM that use the same electron donors (Carlson and Hubert 2019). Furthermore, some lithotrophic NRM can couple the reduction of nitrate to sulfide oxidation, effectively catalyzing the opposite of the souring reaction (Carlson and Hubert 2019). Nitrate reduction can lead to an increase in nitrite, resulting in sulfate reduction being inhibited (Nemati et al. 2001, Greene et al. 2003).

Produced water storage ponds allow temporary retention of a mixture of water, residual hydrocarbons, and organic and inorganic compounds following separation of water from produced oil and gas (Lyman et al. 2018). This strategy allows for produced water to be reused and reinjected into the subsurface to avoid reliance on freshwater. The reuse of produced water involves its storage during a prolonged period before its reuse, and the water can quickly turn sour if not properly maintained (Shi et al. 2023). Common constituents of produced water microbiomes are members of the genera *Halanaerobium, Halomonas, Idiomarina*, and *Marinobacter*, as summarized in Kashani et al. (2024). Members of the genus *Halanaerobium* have been shown to be sulfidogenic (via thiosulfate reduction), while members of the three other genera are notable for potential roles in souring prevention in response to nitrate addition (Suri et al. 2017, Nicoletti et al. 2020). Preventing biofouling and souring within produced water storage ponds is therefore critical to ensuring the integrity of injection wells used in hydraulic fracturing. While the addition of oxidizing agents has been used by industry to remove sulfide and control bacteria, their effect is not consistent (Shi et al. 2023) such that the use of other techniques is required. In light of this, nitrate was added directly to a produced water storage pond to investigate souring control over a 4-week period. Geochemical analyses coupled with amplicon and metagenomic sequencing enabled monitoring a microbial succession within the pond microbiome in response to nitrate addition.

## Materials and methods

### Sample collection and handling

Samples were collected from a produced water storage pond associated with hydraulic fracturing operations in the Permian Basin (TX, USA). The produced water originated from multiple wells, with geochemistry similar to what has been reported previously (Jiang et al. 2022). Water was collected 1 day before nitrate was added (day −1), on the day of the nitrate addition (day 0) and then periodically over the course of 28 days after the nitrate addition. Nitrate dosing to the pond was calculated based on stochiometry related to available carbon sources, with a 20%–30% margin. In total, sampling was performed on days −1, 0, 1, 3, 7, 14, 21, and 28, consisting of collecting 2 l that was frozen immediately and then transported to the laboratory for further analyses.

### Chemical measurements

Samples were thawed and 4-ml aliquots were passed through a 0.2-µm polyethersulfone filters (VWR; Mississauga, Ontario, Canada) and stored at −20°C until analyses. Sulfate, thiosulfate, nitrate, and nitrite levels were assessed by ion chromatography (Dionex ICS-5000) using an analytical column (AS23) with 8 mM Na_2_CO_3_/1 mM NaHCO_3_ eluent at a flow rate of a 1 ml/min. The absence of sulfide production in the pond (personal communications) and the time elapsed during shipment to the laboratory did not allow for confident laboratory-based sulfide measurements. Chromeleon software was used for visualization. Peak area calibration used Na_2_SO_4_ standard solutions. Volatile fatty acid levels were measured using high-performance liquid chromatography in an Ultimate 3000 RSLC system with a 5 mM H_2_SO_4_ mobile phase at a flow rate of 0.6 ml/min and a temperature of 60°C using a Bio-Rad Aminex HPX-87H column. Colleagues at the Canada Excellence Research Chair in Materials Engineering analyzed organic and inorganic carbon levels within each sample. To perform those analyses, a total organic carbon (TOC) analyzer from Shimadzu (Kyoto, Japan) was operated with the following parameters: the flow gas (purified air) is passed at 150 ml/min through an oxidation catalyst-filled combustion tube and heated to 680°C. Results were converted from mg/L to mM concentrations of carbon. Inorganic carbon concentrations were obtained by substracting the organic carbon concentration from the total carbon values.

### DNA extraction and 16S rRNA gene sequencing

For further DNA analyses, all samples were passed through a 0.1-µm polyethersulfone membrane filter using an autoclaved, UV-sterilized filtration unit. A total of 400 ml was filtered for each sample. Filters were stored within Powersoil PowerBead tubes (Qiagen, Hilden, Germany) and kept at −20°C until DNA extraction was performed. Upon thawing, genomic DNA was extracted using the DNeasy PowerSoil extraction kit (Qiagen) following the manufacturer’s instructions. Resulting DNA concentrations were measured using a Qubit fluorometer (Qiagen, Hilden, Germany). DNA was used for both 16S rRNA gene amplicon sequencing (described later) and shotgun metagenomic sequencing (see the “Metagenomic sequencing and analyses” section).

The V4 hypervariable region of the 16S rRNA gene was amplified using primers 515F (GTGYCAGCMGCCGCGGTAA) and 806R (GGACTACNVGGGTWTCTAAT) (Wei et al. 2023). Polymerase Chain Reaction (PCR) with a total volume of 25 µl included: 2 × KAPA HiFi Hot Start Ready Mix (KAPA Biosystems, Wilmington, MA, USA), a final concentration of 0.1 mM for each primer and 7.5 µl of DNA sample. PCR included an initial denaturation at 95°C for 5 min followed by 30 cycles of denaturation at 95°C for 30 s, annealing at 55°C for 45 s and extension at 72°C for 1 min. This was followed by a final extension for 5 min at 72°C. Triplicate PCR products were pooled prior to cleanup and indexing following the manufacturer’s instructions for sequencing using Illumina’s v3 600-cycle (paired-end, 2 × 250 bp) reagent kit on an in-house MiSeq benchtop sequencer (Illumina, San Diego, CA, USA). Prior to sequencing, all DNA extraction blanks and PCR reagent blanks were confirmed to be negative for amplification with the absence of bands on an agarose gel. Quality-controlled reads from eight different samples were merged and subsequently dereplicated to construct an amplicon sequence variant (ASV) table using DADA2 on R (DADA2 2024). Relative abundance calculations were based on the number of unrarefied reads per library, which ranged from 7645 to 102 326 reads. R and GraphPad Prism were used for visualization of the data. Relative abundance cut-offs are indicated in the figure legends (1% in Fig. 2, no cut-off in Fig. 3).

### Metagenomic sequencing and analyses

Shotgun sequencing of genomic DNA was performed on the day 21 sample (Novogene, Sacramento, CA, United States). Adapters were removed, the last 151 bp of the last read was trimmed, PhiX spike-in sequences (PhiX control V3 library, Illumina) were filtered out, and low-quality ends were clipped off using BBDuk. Assembly was performed with Megahit (Li et al. 2015). Reads were mapped using Bowtie2 (Langmead and Salzberg 2012, Li et al. 2015). Binning of the contigs into metagenome-assembled genomes (MAGs) used Metabat (Kang et al. 2015). Quality assessment of the dereplicated bins was performed with CheckM “lineage_wf” (v.1.1.2) with MAG taxonomy assigned using GTDB-Tk “classify_wf” (v.1.5.0) (Parks et al. 2015, Olm et al. 2017, Chaumeil et al. 2020). Only bins with <15% redundancy and >50% completeness were retained for annotation and further study. DRAM (v.1.2) was used to annotate MAGs for functional potential (Shaffer et al. 2020), and phyloFlash allowed for taxonomic identification from the metagenomic reads (Gruber-Vodicka et al. 2020).

## Results

### Microbial activity in the produced water storage pond

**Figure 1.**
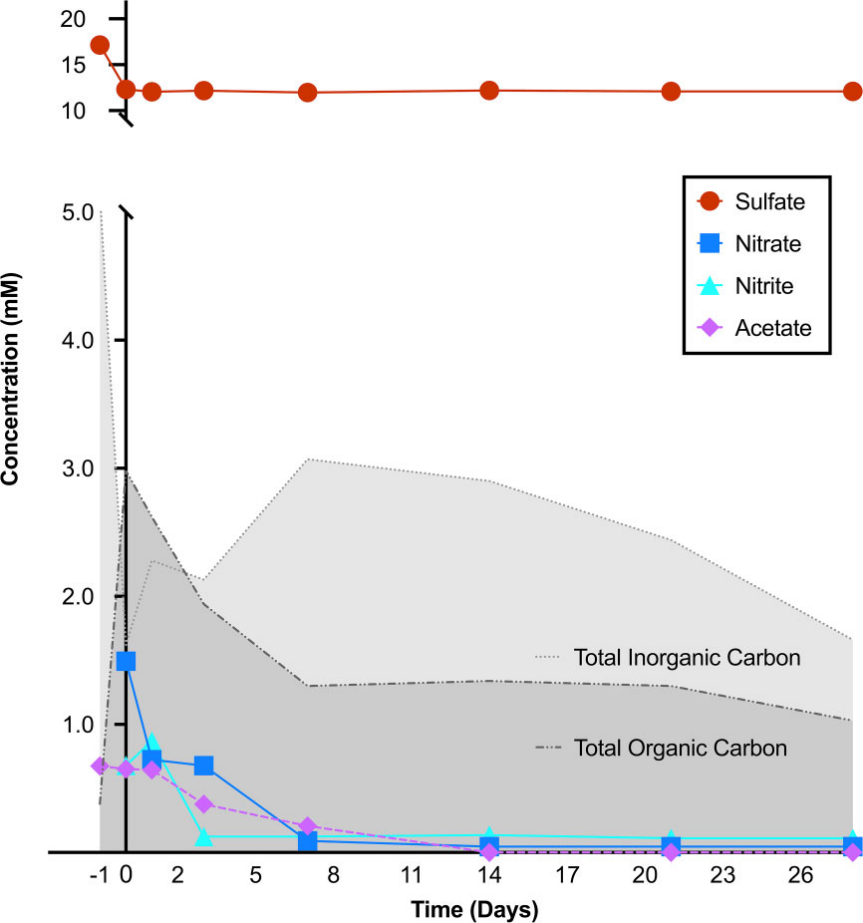
Concentrations of acetate, nitrate, nitrite, and sulfate, as well as total organic carbon and total inorganic carbon, measured within the storage pond over the 4-week monitoring period. Conditions in the pond prior to nitrate injection are plotted at −1 on the x-axis, with day 0 corresponding to the nitrate addition.

Levels of nitrate, nitrite, thiosulfate, and sulfate in pond water were measured to assess the effectiveness of nitrate addition for souring prevention. Initially, a drop in sulfate of 4.9 mM prior to nitrate addition (Fig. 1) was consistent with sulfate reduction and the risk of souring, despite direct evidence of sulfide production not being observed by the facility operator. Following addition of nitrate (day 0), sulfate remained at a constant level, ∼12 mM, with no further sulfate reduction observed throughout the monitoring period. While thiosulfate can also be a precursor to souring, it was not detected in any of the samples suggesting that it was not present or lower than the 0.1 mM detection limit of the ion chromatography method used for analysis.

Nitrate consumption was observed within 1 week of its addition. Upon its addition at day 0, nitrate levels were ∼1.5 mM, and quickly decreased the next day (day 1) to 0.7 mM, eventually reaching 0.1 mM by day 7 (Fig. 1). Nitrite increased initially from 0.7 to 0.9 mM within 24 h of nitrate addition, and then decreased rapidly to reach a concentration of 0.1 mM after 3 days.

Water samples were also tested for volatile fatty acids to assess microbial activity within the pond. Screening for acetate, butyrate, formate, lactate, propionate, and succinate revealed that only acetate could be detected. Acetate levels remained stable at 0.7 mM before and after the initial addition of nitrate, and then started to decrease at 3 days post-addition, dropping to 0.4 mM. Acetate was no longer detected after 14 days. These acetate dynamics were reflected in a general decrease in TOC that was most pronounced over the first 7 days, with a corresponding increase in total inorganic carbon (TIC), consistent with acetate-driven organotrophic nitrate reduction during this period (Fig. 1). TIC then gradually decreased between 7 and 28 days, dropping from 3.1 to 1.7 mM. These changes were more gradual than the sharp changes in TOC and TIC before and after nitrate addition (days −1 and 0).

### Pond water microbial community composition

**Figure 2.**
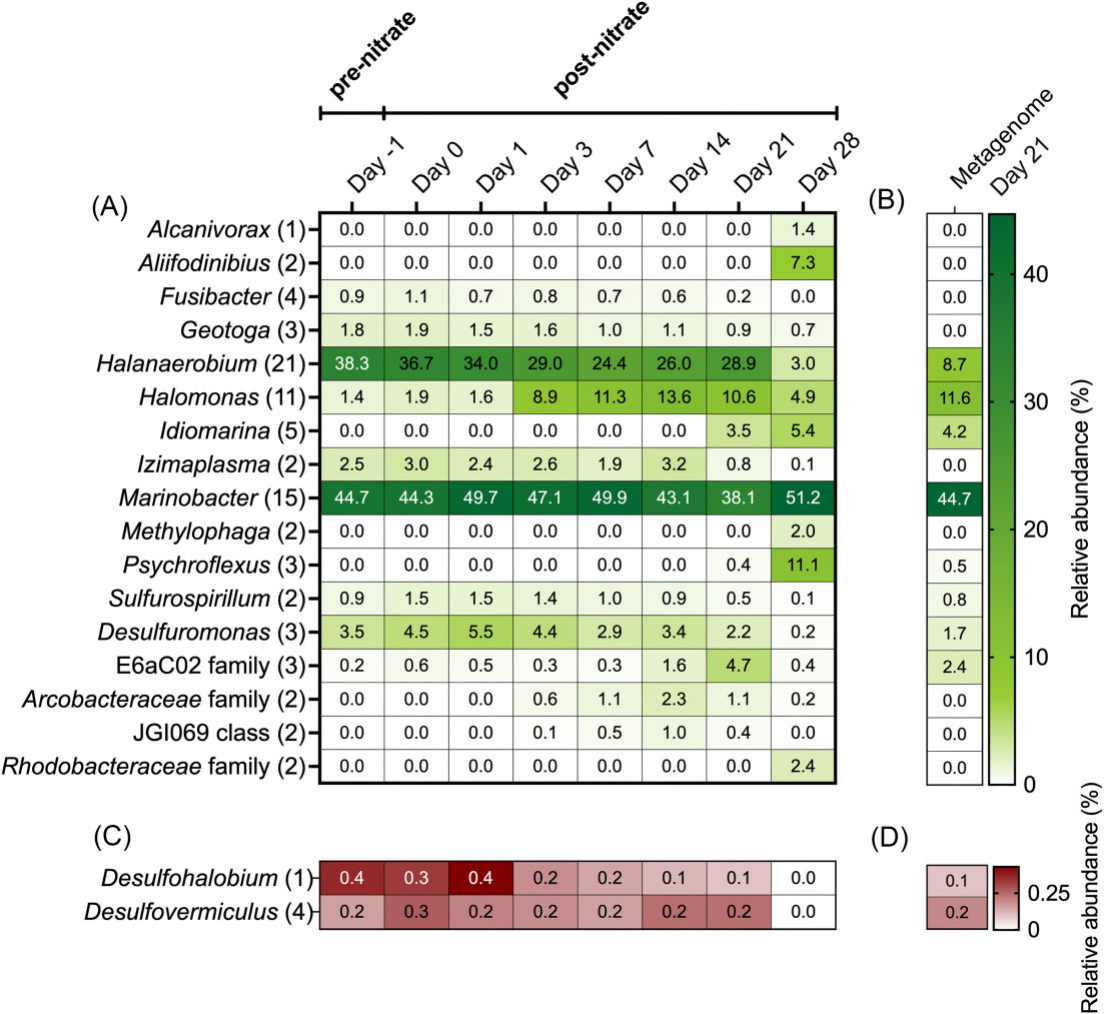
Microbial community composition analyzed by (A) 16S rRNA gene amplicon sequencing showing the relative abundance of ASVs (only ASVs detected above 1% in at least one sample are included) throughout the 4-week period with the total number of unique ASVs within each lineage noted in parentheses (see Table S1 for more information), and (B) relative abundance of the same lineages within metagenome reads obtained from the day 21 DNA sample based on phyloFlash taxonomic assignments. The bottom part of the figure shows that putative sulfate-reducing microbial groups were all below 1% in amplicon libraries; thus, they are plotted separately (C) and correspond to similarly low relative abundances in the day 21 metagenome (D).

Prior to nitrate addition (−1 days), the pond water microbial community was characterized by *Halanaerobium* and *Marinobacter* ASVs with relative abundances of 38% and 45%, respectively (Fig. 2A; Table S1). While a decrease in the relative abundance of *Halanaerobium* was observed over time following nitrate addition, levels of *Marinobacter* remained relatively high. Other genera also decreased in abundance in the days following nitrate addition, notably *Fusibacter, Geotoga*, and *Izimaplasma*. Groups showing an increase in abundance included *Halomonas, Sulfurospirillum, Desulfuromonas*, and members of the E6aC02 family. *Halomonas* exhibited the most notable enrichment in abundance during the monitoring period, increasing from 0.4% (pre-nitrate addition) to 13.6% after 14 days. Similarly, lineages that were below detection in early stages and then increased in relative abundance in later stages of the monitoring period were *Alcanivorax, Aliifodinibius, Idiomarina, Methylophaga, Psychroflexus*, members of the JGI069 class, and members of the *Rhodobacteraceae* and *Arcobacteraceae* families.

Despite the apparent sulfate consumption observed prior to nitrate addition to the storage pond (Fig. 1), putative SRM were detected in low relative sequence abundance in the amplicon libraries. Fig. 2C shows SRM reaching maximum levels of 0.4%–0.6% in the pond during an early stage in the monitoring. This signal declined below detection limit by the end of the monitoring period (i.e. <0.007% relative sequence abundance based on 13 967 reads in the day 28 amplicon library).

To better understand how nitrate metabolism prevents souring within the storage pond, metagenomic sequencing was performed after 21 days, i.e. the point at which amplicon sequencing suggested high representation of putative nitrate-reducing microorganisms (NRM) such as *Halomonas* and *Idiomarina* (Fig. 2A and B). Metagenomic reconstruction resulted in 19 MAGs with <15% redundancy and >50% completeness (Table 1). Out of the 13 taxonomic groups of ASVs identified to the genus level that were abundant (>1%) in the amplicon libraries (Fig. 2A), 8 were observed at similarly high abundance in quality-controlled metagenomic reads using phyloFlash (Fig. 2B). The most notable exception to this broad congruence is the discrepancy of *Halanaerobium* relative abundance being 28.9% based on amplicon sequencing compared to 8.7% in the metagenomic library. Metagenomic analysis also suggests that the decreasing abundance of *Halanaerobium* over 21 days may be more rapid and pronounced than the amplicon time course analysis reveals (Fig. 2).

**Table 1.** Quality assessment and taxonomic profile of MAGs obtained from the pond microbiome at day 21.

MAG ID	Genome size (bp)	Contigs (*n*)	N50	C^a^ (%)	R^b^ (%)	SH^c^ (%)	GC (%)	Domain	Phylum	Class	Order	Family	Genus	Species
1	3 081 019	56	8 222	60	12	85.71	67	*Bacteria*	*Desulfobacterota*	*Desulfuromonadia*	*Desulfuromonadales*	*Desulfuromonadaceae*		
2	2 021 602	51	8 359	57	12	71.43	69	*Bacteria*	*Bacteroidota*	*Rhodothermia*	*Balneolales*	M1805		
3	4 165 509	64	8 902	89	13	83.08	70	*Bacteria*	*Bacteroidota*	*Bacteroidia*	*Bacteroidales*	S143-32		
4	3 724 211	722	9 888	64	8	40	67	*Bacteria*	*Proteobacteria*	*Gammaproteobacteria*	*Nitrococcales*	*Nitrococcaceae*	*Arhodomonas*	*A. aquaeolei*
5	3 554 629	145	16 674	95	3	8.33	38	*Bacteria*	*Deferribacterota*	*Deferribacteres*	*Defferibacterales*	*Flexistipitaceae*	*Flexistipes*	
6	2 047 885	113	22 053	95	2	72.73	46	*Bacteria*	*Proteobacteria*	*Gammaproteobacteria*	*Halothiobacillales*	*Halothiobacillaceae*	*Guyparkeria*	
7	1 002 731	529	3 557	55	0	100	39	*Bacteria*	*Proteobacteria*	*Gammaproteobacteria*	*Pseudomonadales*	*Halomonadaceae*	*Halomonas*	
8	1 730 030	324	11 773	59	4	100	60	*Bacteria*	*Proteobacteria*	*Gammaproteobacteria*	*Pseudomonadales*	*Halomonadaceae*	*Halomonas*	*H. ventosae*
9	3 158 056	519	6 131	81	7	50	29	*Bacteria*	*Proteobacteria*	*Gammaproteobacteria*	*Pseudomonadales*	*Halomonadaceae*	*Halomonas*	*H. alimentaria*
10	2 246 803	213	13 459	93	2	33.33	47	*Bacteria*	*Proteobacteria*	*Gammaproteobacteria*	*Enterobacterales*	*Alteromonadacea*	*Idiomarina*	*I. batica*
11	1 455 637	92	37 523	59	1	100	51	*Bacteria*	*Firmicutes*	*Bacilli*	*Izemoplasmatales*	*Izemoplasmataceae*	M55B118	
12	2 382 032	718	9 790	97	2	66.67	33	*Bacteria*	*Firmicutes*	*Halanaerobiia*	*Halanaerobiales*	M55B127	M55B127	
13	3 902 706	218	30 267	83	9	14.29	60	*Bacteria*	*Proteobacteria*	*Gammaproteobacteria*	*Pseudomonadales*	*Oleiphilaceae*	*Marinobacter*	*M. persicus*
14	1 388 483	60	23 046	93	1	100	45	*Bacteria*	*Proteobacteria*	*Gammaproteobacteria*	*Pseudomonadales*	*Oleiphilaceae*	*Marinobacter*	*M. segnicrescens*
15	2 244 727	197	36 013	93	4	25	67	*Bacteria*	*Proteobacteria*	*Gammaproteobacteria*	*Pseudomonadales*	*Oleiphilaceae*	*Marinobacter*	*M. sp014359475*
16	1 550 490	347	36 990	76	1	0	38	*Bacteria*	*Bacteroidota*	*Bacteroidia*	*Flavobacterales*	*Flavobacteriaceae*	*Psycrhoflexus*	*P. salarius*
17	4 026 439	349	109 131	83	0	0	44	*Bacteria*	*Bacteroidota*	*Bacteroidia*	*Bacteroidales*	PWNQ01	PWNQ01	
18	2 193 566	276	16 205	66	0	0	59	*Bacteria*	*Campylobacterota*	*Campylobacteria*	*Campylobacterales*	*Sulfurospirillaceae*	*Sulfurospirillum*	
19	3 918 585	308	98 410	99	2	0	41	*Bacteria*	*Campylobacterota*	*Campylobacteria*	*Campylobacterales*	*Sulfurovaceae*	TCS-49	

a C, completeness.

b R, redundancy.

c SH, strain heterogeneity.

**Figure 3.**
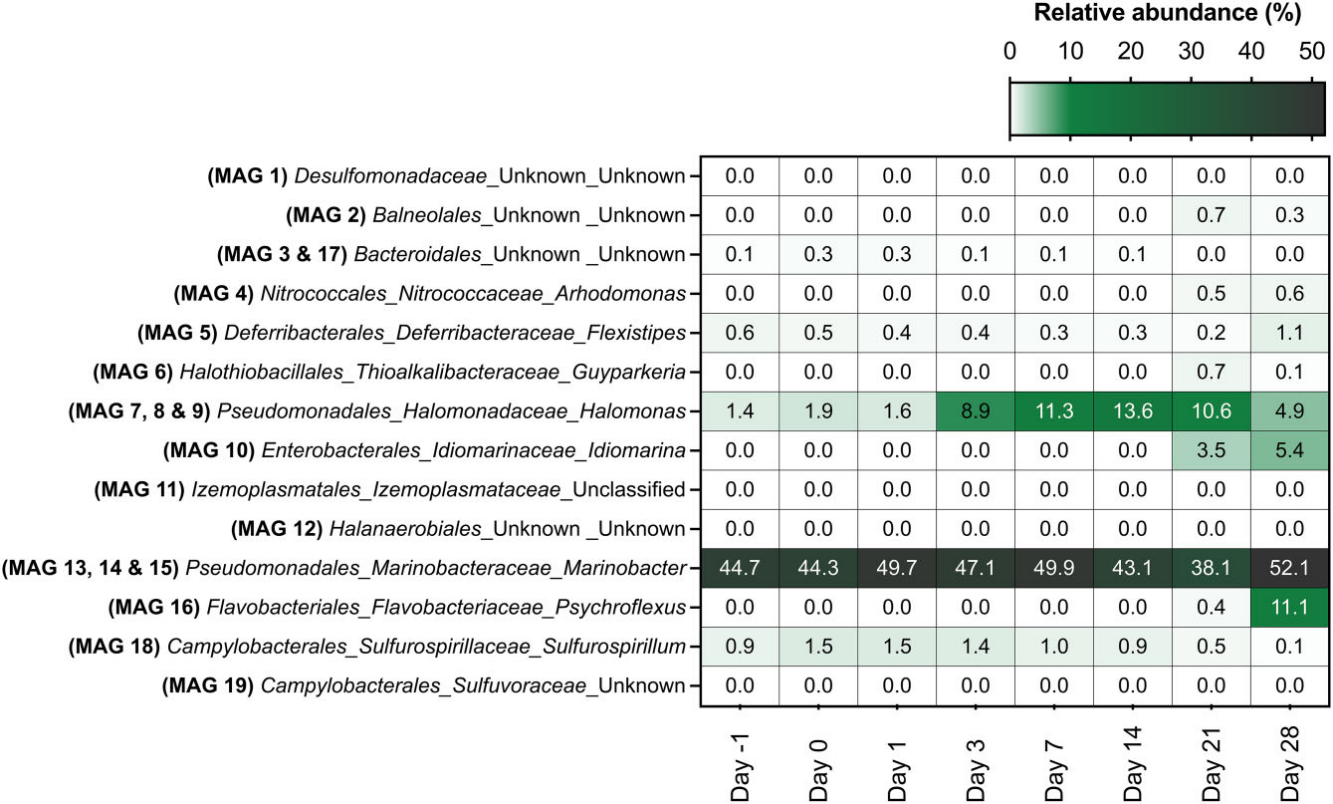
Relative abundance of ASVs belonging to taxonomic lineages that match MAGs throughout the 4-week monitoring period. Row labels indicate order, family, and genus taxonomies for the corresponding MAGs.

MAGs for which taxonomy was assigned with GTDB-Tk enabled tracking corresponding ASVs in the amplicon libraries in order to link metabolic potential from MAG analysis with temporal dynamics in the pond microbiome from ASV analysis (Fig. 3). Groups experiencing early enrichment after nitrate addition (days 0–7) included *Bacteroidales* S143-32 (MAG 3, 17), *Marinobacter* (MAG 13, 14, 15), and *Sulfurospirillum* (MAG 18). *Flexistipes* (MAG 5) decreased in relative abundance between days 0 and 21, but increased from 0.2% to 1.1% between days 21 and 28 (Fig. 3). Later in the incubation (days 14–21), amplicon sequencing highlights increases in *Balneolales* (MAG 2), *Arhodomonas* (MAG 4), *Guyparkeria* (MAG 6), *Halomonas* (MAG 7, 8, 9), *Idiomarina* (MAG 10), and *Psychroflexus* (MAG 16).

### Metabolic pathway reconstruction within MAGs enriched following nitrate addition

#### Nitrate reduction

**Figure 4.**
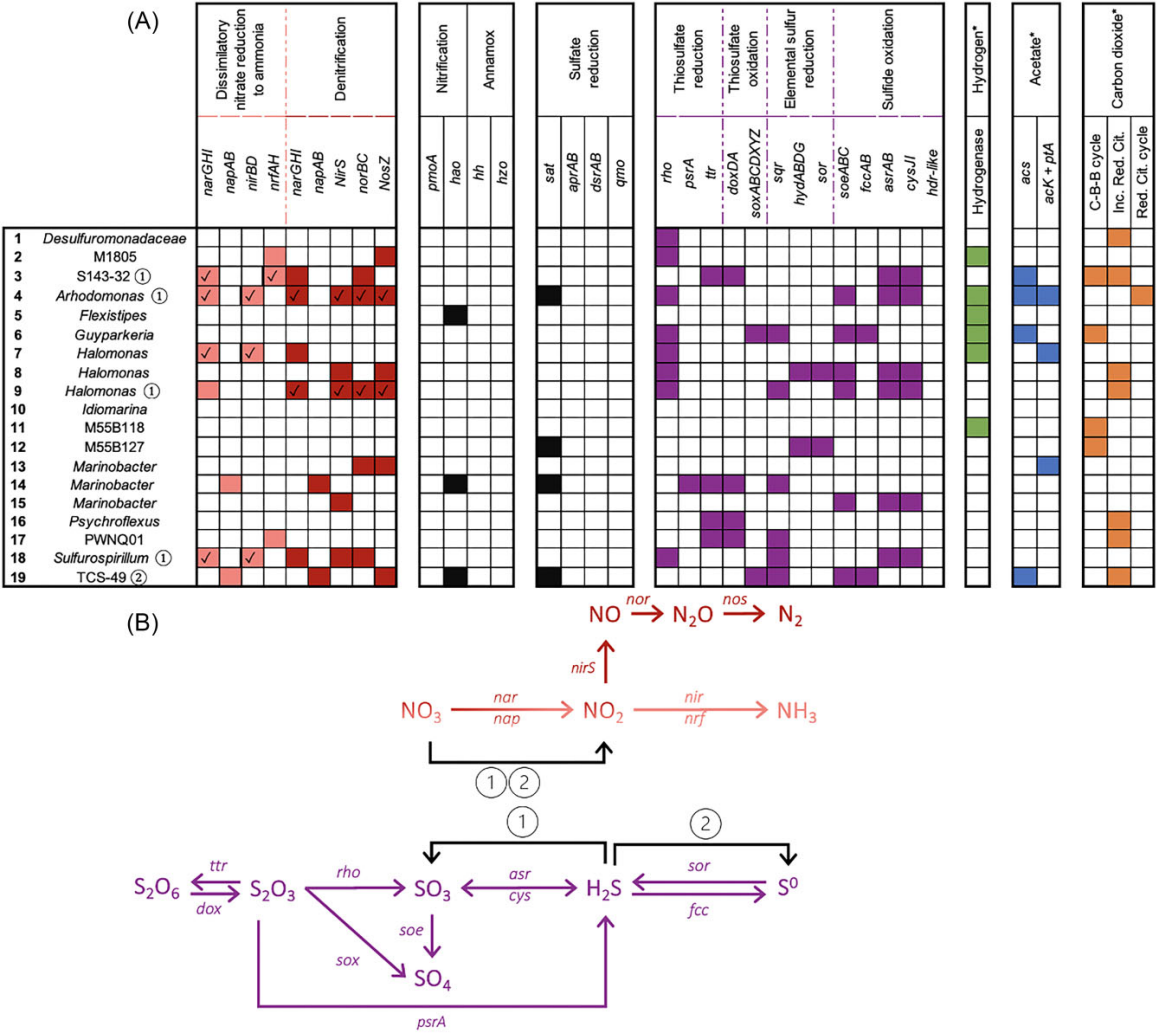
Metabolic potential related to (A) nitrogen, sulfur, hydrogen, and carbon metabolism among MAGs (numbered and listed at left) reconstructed from the storage pond microbiome 21 days after nitrate injection. Complete pathways for nitrate metabolism have a checkmark. Nitrate reduction and sulfur cycling pathways (B) illustrate the potential for nitrate reduction to nitrite to be coupled to sulfide oxidation to sulfite (denoted by 1) or sulfide oxidation to elemental sulfur (denoted by 2), with genomes harboring these combinations noted in (A) with ① and ② symbols beside the MAG names on the left. More detailed gene annotations are available in the Supplementary Material (Figure S1).

MAGs of interest with respect to nitrogen metabolism (Fig. 4) are affiliated with *Arhodomonas aquaeolei* (MAG 4), *Halomonas* (novel species; MAG 7), *Halomonas alimentaria* (MAG 9), *Marinobacter segnicrescens* (MAG 14) and *Sulfurospirillum* (novel species; MAG 18), *Bacteroidales* (novel family S143-32; MAG 3), and *Sulfurovaceae* (novel genus TCS-49; MAG 3). Genes for the reduction of nitrate to dinitrogen (denitrification) were observed in *A. aquaeolei* (MAG 4) and *H. alimentaria* (MAG 9; Fig. 4). Potential for dissimilatory nitrate reduction to ammonia (DNRA) was observed in *Bacteroidales* (MAG 3), *A. aquaeolei* (MAG 4), *Halomonas* (MAG 7), and *Sulfurospirillum* (MAG 18; Fig. 4). Additional potential for nitrate reduction only to nitrite (i.e. *narGHI* or *napAB* genes) was also observed in *M. segnicrescens* (MAG 14) and *Sulfuvoraceae* (MAG 19) with genes for further nitrite reduction (complete denitrification or DNRA) potentially not captured (Fig. 4) due to these MAGs having 93%–99% estimated completeness (Table 1).

#### Lithotrophic metabolism

Genes involved in the oxidation of sulfide to sulfite (*asrAB* and *cysJI*) were seen within *Bacteroidales* S143-32 (MAG 3), *A. aquaeolei* (MAG 4), *H. alimentaria* (MAG 9), and *Sulfurospirillum* (MAG 18). S143-32 represents a family (MAG 3) within the *Bacteroidales* order reported at very low relative abundance within the metagenome (0.2%). Potential for sulfide oxidation to elemental sulfur (*fccAB* genes) was observed only within *Sulfurovaceae* TCS-49 (MAG 19) (Fig. 4). Genes for hydrogen metabolism (*hypF*) were found within *Balneolales* (MAG 2), *Guyparkeria* (MAG 6), *Halomonas* (MAG 7), and *Izemoplasmataceae* (MAG 11), and genes for [NiFe] hydrogenases were found in *A. aquaeolei* (MAG 4), *Flexistipes* (MAG 5), and *Halomonas* (MAG 7).

#### Carbon metabolism

Internalization of acetate can be mediated by the cation/acetate symporter encoded by the *actP* gene, found within *Balneolales* M1805 (MAG 2), *Bacteroidales* S143-32 (MAG 3), *A. aquaeolei* (MAG 4), *Guyparkeria* (MAG 6), *Halomonas* (MAG 7), *Halomonas ventosae* (MAG 8), *Idiomarina bentica* (MAG 10), *Halanaerobiales* M55B127 (MAG 12), *Marinobacter persicus* (MAG 13), *Marinobacter* (MAG 15), *Psychroflexus solarius* (MAG 16), and *Sulfurospirilum* (MAG 18; Figure S1). Of those MAGs, genes for acetate metabolism via an acetyl-CoA synthetase are present within *Bacteroidales* S143-32 (MAG 3), *A. aquaeolei* (MAG 4), *Guyparkeria* (MAG 6), and *Sulfurovaceae* TCS-49 (MAG 19). Genes for acetate kinase and phosphate acetyltransferase were found within *A. aquaeolei* (MAG 4), *Halomonas* (MAG 7), and *M. persicus* (MAG 13; Figure S1).

Carbon fixation via the Calvin–Benson–Bassham cycle is evidenced in genomes of *Bacteroidales* S143-32 (MAG 3), *Guyparkeria* (MAG 6), *Izemoplasmataceae* M55B118 (MAG 11), and *Halanaerobiales* M55B127 (MAG 12). Nearly complete sets of genes for the incomplete reductive citrate cycle were found within MAG 1 *Desulfuromonadaceae* (MAG 1), *Bacteroidales* S143-32 (MAG 3), *H. ventosae* (MAG 8), *H. alimentaria* (MAG 9), *Psychroflexus salarius* (MAG 16), *Bacteroidales* PWNQ01 (MAG 17), and *Sulfurovaceae* TCS-49 (MAG 19). *Arhodomonas aquaeolei* (MAG 4) was the only genome with a nearly complete set of genes for the reductive citrate cycle (Figure S1).

## Discussion

Nitrate addition for souring control has been studied in different contexts in oil systems, most often in laboratory settings (Hubert and Voordouw 2007, Suri et al. 2017, Kamarisima et al. 2018, Wu et al. 2018, Nicoletti et al. 2022). Here, nitrate amendment directly to a produced water storage pond prevented the increase of SRM in a topsides setting (Fig. 2), and sulfate levels remained stable (Fig. 1). The monitoring period of 28 days mimicks the period to which the produced water usually sits in the storage pond before being reused for hydraulic fracturing operations. In addition to preventing souring in the pond, topsides removal of organic electron donors helps to minimize reservoir souring following reinjection of this water into the subsurface. Nitrate levels in the pond decreased in the days following its addition, coinciding with nitrite detection, consistent with the activity of NRM. Bacterial lineages associated with nitrate metabolism increased in relative abundance over the first 8 days of the monitoring period (*Arhodomonas, Halomonas*, and *Sulfurospirillum*; Figs 2B and 3), and five different MAGs with nitrate-related metabolism were recovered. Accordingly, sulfate levels remained stable after nitrate was added to the pond (Fig. 1), highlighting the efficacy of nitrate addition for preventing souring in surface storage pond settings.

The transience of nitrite accumulation in the pond indicates that nitrate reduction to nitrogen (denitrification) or ammonia (DNRA) achieved the observed souring control. Biocompetitive exclusion implies that NRM utilize electron donors that would otherwise be available for SRM to couple to sulfide production. In principle, DNRA achieves greater souring control by this mechanism, since it is an eight-electron transfer (NO_3_^−^ → NH_4_^+^) compared to denitrification being a five-electron transfer (NO_3_^^−^^ → N_2_) and thus consumes more substrate per nitrate reduced. Acetate is a common electron donor used by sulfate-reducers (Skyring 1988, Glombitza et al. 2015) as well as by many organotrophic nitrate-reducing bacteria (Kłodowska et al. 2018). MAGs from the storage pond with potential to use acetate and reduce nitrate were affiliated with *A. aquaeolei, Bacteroidales* S143-32, and *Sulfurovaceae* TCS-49 (Figs 2, 3, and 4). *Arhodomonas aquaeolei* is reported to be a halophile that was first isolated from a petroleum reservoir in 1993 and reportedly has potential for DNRA and denitrification (Adkins et al. 1993). *Bacteroidales* MAG 3 belongs to the novel family S143-32 that was first isolated from a hydrothermal vent (Reysenbach et al. 2020), but has not been extensively investigated. *Sulfurovaceae* TCS-49 has genes for acetate and nitrate metabolism, and members of this lineage are well known for using reduced sulfur compounds as electron donors. The presence of genes involved in sulfur compound oxidation in *Sulfurovaceae* MAG 19 indicates the potential for souring control to alternatively occur via direct removal of sulfide.

Indeed, sulfide generation (souring) can also be reversed by lithotrophic nitrate-reducers that utilize sulfide as an electron donor. Pathways for both sulfide oxidation to sulfite and nitrate reduction to nitrogen (denitrification) were found within *A. aquaeolei* (MAG 4) and *H. alimentaria* (MAG 9) (Figs 3 and 4). While the relative abundance of *Halomonas* started increasing at day 4 as nitrate levels in the storage pond were dropping (Figs 1 and 3), *Halomonas* sequence abundance in the pond continued to increase once nitrate was below detection limits. *Halomonas alimentaria* has been shown to be involved in hydrocarbon biodegradation (Olajire and Essien 2014) and has also been implicated in denitrification coupled to souring control (González-Domenech et al. 2010, Perman et al. 2022). These earlier observations could also be due to lithotrophic sulfide oxidation by members of this genus, based on the pattern observed here. Genes for both sulfide oxidation and DNRA were found within *Bacteroidales* S143-32 (MAG 3) and *Sulfurospirillum* (MAG 18). Relative abundances of both of these groups increased in the early stage of monitoring when nitrate was still detected in the pond, then slowly decreased as nitrogen compounds and organic carbon levels declined (Figs 1, 3, and 4).

Microorganisms need not be limited to only one metabolic mechanism for contributing toward souring control. *Sulfurospirillum* spp. have been shown to control souring in laboratory settings owing to the ability to couple DNRA to either organotrophic or lithotrophic metabolism (Hubert and Voordouw 2007, Ross et al. 2016, Hillman et al. 2022). While this potential was not uncovered in the *Sulfurospirillum* MAGs discovered in this study, three other MAGs possessed genes for both organotrophic and lithotrophic metabolism as well as genes for nitrate reduction. These belonged to *A. aquaeolei* (MAG 4), *Sulfuvoraceae* TCS-49 (MAG 19), and *Bacteroidales* S143-32 (MAG 3). Despite these wide arrays of metabolic genes, the increase in relative abundance within the initial monitoring stage for MAG 3 (S143-32) suggests its role in the pond was organoheterotrophic nitrate reduction based on the observed acetate consumption being coupled with nitrate reduction (Figs 1, 3, and 4), thereby preventing acetate-driven sulfate reduction (i.e. biocompetitive exclusion of SRM by NRM). The increase in relative abundance of ASVs affiliated with *A. aquaeolei* (MAG 4) during later stages of the monitoring period may indicate a role in autotrophic carbon fixation (Figs 3 and 4), given the decline in TIC measured at the same time (Fig. 1).

Monitoring the biogeochemistry, microbial diversity and genomic potential of a nitrate-treated storage pond revealed enrichment of nitrate-reducing populations and mitigation of sulfate reduction over a 4-week period. Putative nitrate-metabolizing organisms, *Arhodomonas, Halomonas*, and *Sulfurospirillum*, have been found in other produced water studies (Hubert et al. 2007, Mnif et al. 2009, Mbow et al. 2024). Potential for coupling nitrate reduction (through DNRA or denitrification) to either biocompetitive exclusion by organotrophic nitrate-reducers or sulfide oxidation by lithotrophic nitrate reducers, as well as inhibition of sulfate-reducing bacteria via nitrite production, is widely reflected in the genomic potential of the pond microbiome that emerged following nitrate addition. This study demonstrates nitrate treatment as a promising strategy for souring management in topsides oil field-produced water storage ponds. For industry operations, these findings highlight the importance of sufficient topsides nitrate addition to storage ponds in order to exhaust carbon sources that would otherwise be available for sulfidogenic bacteria to metabolize after nitrate is fully consumed in the storage pond. Additional nitrate dosing may thus be needed if produced water is stored in topsides pond environments for a prolonged period (Shi et al. 2023).

## Supplementary Material

fiaf041_Supplemental_Files

## Data Availability

All amplicon and metagenomic data are available under the National Genomics Data Center bioproject PRJCA024061. All codes and software used can be found at the following address: https://github.com/gabriellescheffer/Metagenomics_DigitalResearchAllianceCanada.
