# Supplementary material for: Nitrate-reducing microorganisms prevent souring of an oil field produced water storage pond
Source: FEMS Microbiol Ecol. 2025 Apr 28;101(5):fiaf041. doi: 10.1093/femsec/fiaf041 (PMC12047076; doi:10.1093/femsec/fiaf041)
Supplement: fiaf041_Supplemental_Files [file GS_Figure S1_FEMS.pdf › GS_Figure S1_FEMS.pdf]

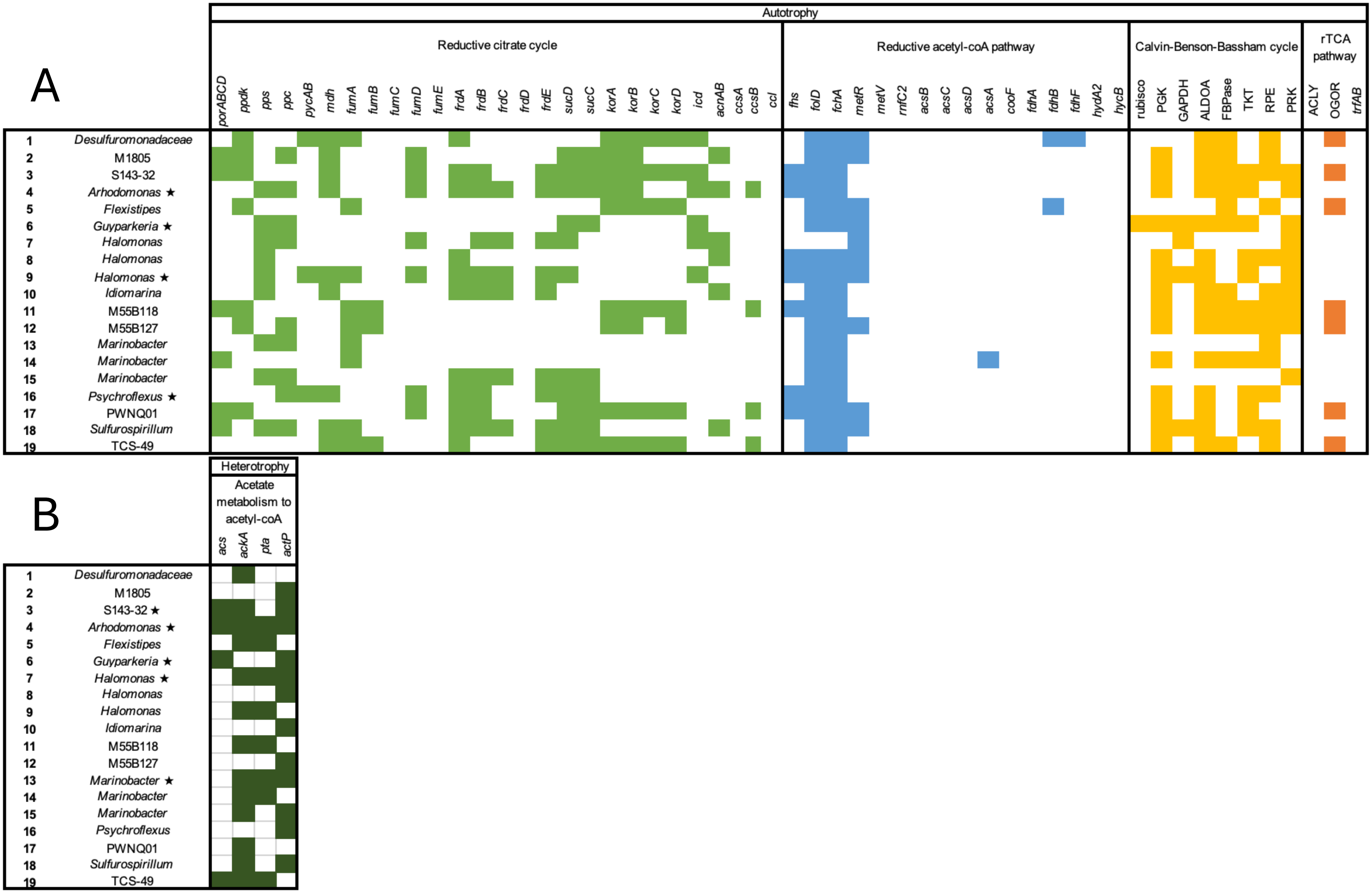

Supplementary Figure 1: Detailed genes for (A) autotrophic metabolism including the reductive citrate cycle (light green), the reductive acetyl-coA pathway (blue), the Calvin-Benson-Bassham cycle (yellow) and the rTCA pathway (orange), and for (B) heterotrophic metabolism including acetate metabolism to acetyl-coA (dark green). The various colors under each gene in the table indicates their presence for each MAG.
